# ABO blood group is not a predictive factor for the amount of early opioid consumption in postanesthesia care unit: a prospective cohort study in 3,316 patients

**DOI:** 10.1186/s12871-022-01583-y

**Published:** 2022-02-16

**Authors:** Sasikaan Nimmaanrat, Alan Geater, Prae Plunsangkate, Laortip Saewong, Orarat Karnjanawanichkul, Thavat Chanchayanon, Ngamjit Pattaravit

**Affiliations:** 1 Department of Anesthesiology, Faculty of Medicine, Prince of Songkla University, Hat Yai, Songkhla, 90110 Thailand; 2 Epidemiology Unit, Faculty of Medicine, Prince of Songkla University, Hat Yai, Songkhla, 90110 Thailand

**Keywords:** ABO blood group, Predictive factors, Postoperative, Opioid requirement, Postanesthesia care unit, PACU

## Abstract

**Background:**

Immediate postoperative pain in the postanesthesia care unit (PACU) is common. Titration of opioid is the most popular strategy for controlling early postoperative pain. ABO blood group has been found to be associated with pain perception. We aimed to find the factors including ABO blood group for predicting the amount of opioid requirement in PACU.

**Methods:**

This prospective cohort study was performed in 3316 patients who underwent various kinds of anesthetic techniques and received immediate postoperative care in PACU. Preoperative, intraoperative and PACU data were collected. A directed acyclic graph (DAG) representing the hypothesized causal pathways of preoperative, intraoperative and PACU data were compiled prior to conducting multinomial logistic regression analysis. Statistical significance in all models was defined as a *P*-value < 0.05.

**Results:**

Female sex, body mass index, preoperative use of gabapentin, preoperative anxiety score, anesthetic techniques, type of operation, amount of consumed intraoperative opioids, intraoperative use of paracetamol, parecoxib and ondansetron, duration of anesthesia and surgery, amount of blood loss, pain upon PACU arrival, use of paracetamol and parecoxib in PACU were found to be factors influencing amount of opioid consumption in PACU. ABO blood group was not associated with early postoperative opioid requirement.

**Conclusions:**

A significant number of factors are related to amount of opioid requirement in PACU. Some factors can be adjusted to provide better pain relief in early postoperative period. However, ABO blood group is not identified to be a predictive factor for early postoperative opioid consumption in PACU.

## Introduction

After surgery, the majority of patients spend their immediate postoperative period in a postanesthesia care unit (PACU). A significant number of them first experience postoperative pain in PACU requiring analgesic treatment. Almost half reported moderate or severe pain [1, 2]. Even though the use of non-opioids has been recommended as an important component of multimodal analgesic regimen for postoperative pain control in PACU [3], opioids are still widely used because they provide reliably fast onset and effective outcome. The aim of opioid administration is to provide the maximum analgesia with no intolerable unwanted consequences. Most of opioid-related side effects depend on the dose, including nausea and vomiting, excessive sedation and respiratory depression, needing close observation [4] especially in those who recently awake from anesthesia.

It has been known that there is a wide inter-individual variability in opioid demand. Race (Caucasian), emergency operation, major operative procedures, operation time longer than 100 min and pain score on arriving to PACU have been found to be independent predictive factors of morphine consumption in patients undergoing various non-cardiac surgery under general anesthesia [5].

Genetic factors such as sex have been demonstrated to be associated with pain sensitivity and analgesic response [6]. ABO blood group is one genetic factor and it has been shown to be associated with various medical illnesses such as von Willebrand disease [7], coronary artery disease [8] and cancer [9]. There are conflicting results on whether or not ABO blood group is associated with pain. Simoni et al., demonstrated that participants with blood group AB displayed the strongest conditioned pain modulation effect and blood group B exhibited the lowest mechanical pain sensitivity [10]. Blood group O has been found to be one of the factors affecting post-cesarean pain [11]. On the other hand, different blood group patients undergoing anterior cruciate ligament reconstruction did not show different postoperative analgesic consumption [12]. Nimmaanrat et al., performed a retrospective study in 1530 patients to examine the association of ABO blood group and postoperative opioid consumption within the first 24 h after cesarean delivery. They found that ABO blood group is not a predictive factor for opioid requirement within the first 24 h following cesarean section [13].

We conducted this prospective cohort study in 3316 adult patients mostly scheduled for elective surgery in a tertiary referral medical school with the primary objective to identify the association of ABO blood group and the amount of early postoperative opioid requirement in PACU. Our secondary objective was to identify factors associated with the amount of opioid consumption in PACU. By recognizing patients at risk for high opioid requirement in PACU, it may be possible to offer more effective pain management for them to be more comfortable in the early postoperative phase. Moreover, studies to assess the benefits of a personalized pain relief strategy based on ABO blood group or other factors is warranted.

## Methods

This study followed Strengthening the Reporting of Observational Studies in Epidemiology (STROBE) checklist and was approved by the Ethics Committee of the Faculty of Medicine, Prince of Songkla University, Thailand (REC Number: 61–115–8-1) on 9 May 2018. This study involved no more than minimal risk to subjects so the patient consent was waived by the Ethics Committee of the Faculty of Medicine, Prince of Songkla University. The data were anonymized, maintained with confidentiality and in compliance with the Declaration of Helsinki.

### Patients

Patients scheduled for any elective operation and expected to receive care in the PACU at Songklanagarind Hospital, Hat Yai, Songkla, Thailand were enrolled into this prospective cohort study. Exclusion criteria included patients who were unable to communicate and scheduled for emergency surgery.

### Data collection

Data were recorded on a standard case record form including (A) patient characteristics [ABO blood group, gender, age, race, American Society of Anesthesiologists (ASA) physical status classification, weight, height, body mass index (BMI), in-patient/out-patient, smoking], (B) preoperative data [premedication with benzodiazepine, preoperative use of gabapentin/pregabalin, preoperative anxiety and pain scores using a 0–10 verbal numerical rating scale (VNRS)], (C) anesthetic-related data [anesthetic techniques, amount of intraoperative opioid usage (morphine milligram equivalent - MME), intraoperative usage of non-opioid analgesics, duration of anesthesia], (D) surgical-related data (type of operation, reoperation at the same site, duration of surgery, blood loss) and (E) PACU-related data [pain (VNRS) upon arrival and discharge, amount of opioid usage (MME), usage of non-opioid analgesics, postoperative nausea and vomiting (PONV), length of stay in PACU].

### Sample size calculation

Over the period in which patient data were collected (September 2018–March 2019), the data were available for at least 2533 patients. It was estimated that overall about 11% of patients required opioid at a level of > 5 mg MME and that the proportion of patients of group O requiring such high levels be less than that of non-O patients. With a known ratio of O to non-O patients of approximately 2:3, the sample size would have a power of 80% to detect a difference in proportions requiring > 5 mg MME of 8.8 and 20%, respectively in group O and non-O patients with a type I error of 0.5.

### Statistical analysis

**Fig. 1:**
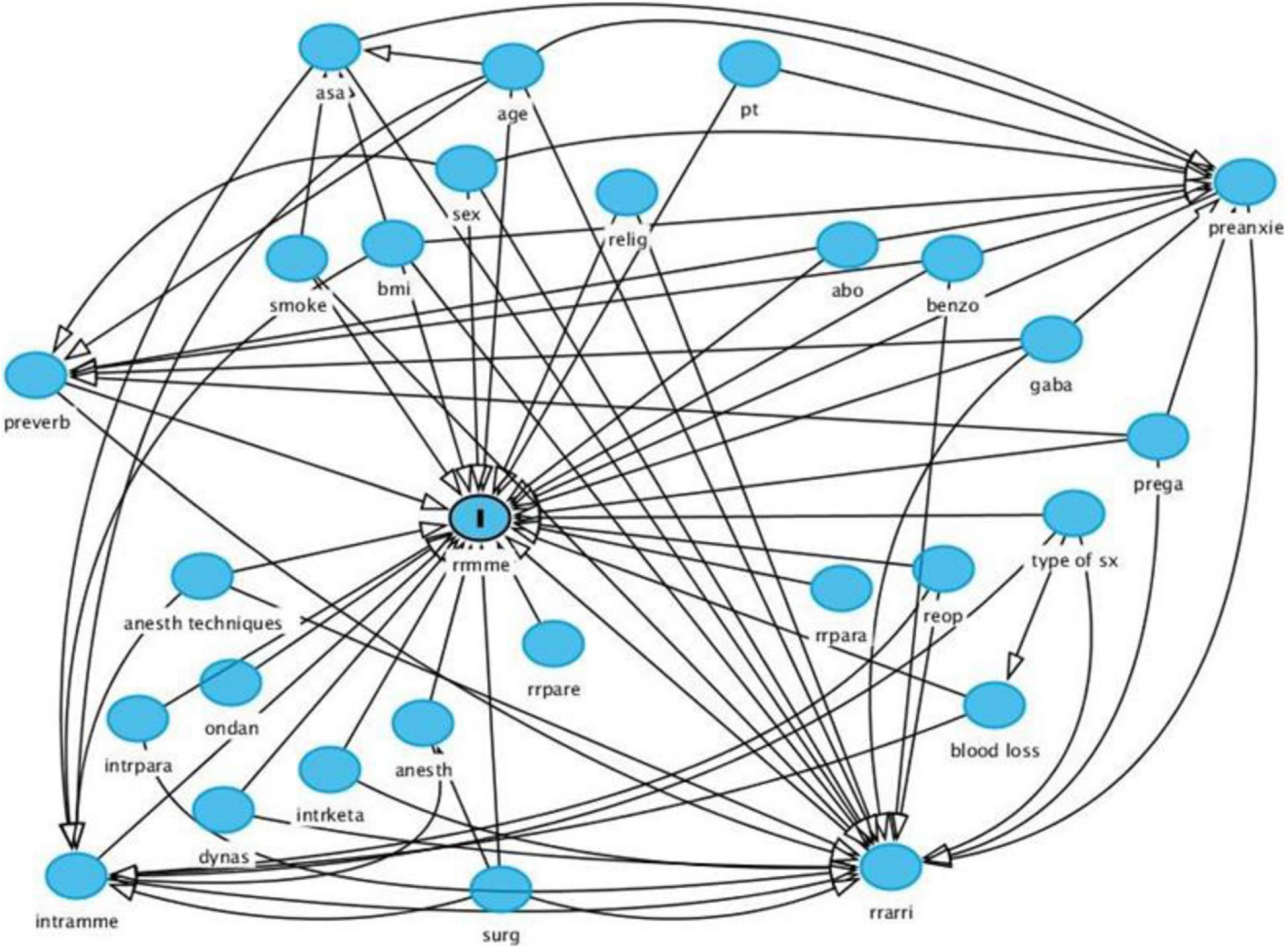
Directed Acyclic Graph (DAG) demonstrating the correlation of each variable. Abbreviations: abo, ABO blood group; anesth, duration of anesthesia; anesth techniques, anesthetic techniques; asa, American Society of Anesthesiologists; benzo, premedication with benzodiazepine; bmi, body mass index; dynas, intraoperative use of parecoxib; gaba, preoperative use of gabapentin; intramme, intraoperative use of morphine milligram equivalent; intrketa, intraoperative use of ketamine; intrpara, intraoperative use of paracetamol; ondan, intraoperative use of ondansetron; preanxie, preoperative anxiety score; prega, preoperative use of pregabalin; preverb, preoperative pain score; pt, in or out patient status; relig, religion; reop, reoperation; rrarri, pain score upon postanesthesia care unit arrival; rrmme, morphine milligram equivalent in postanesthesia care unit; rrpara, use of paracetamol in postanesthesia care unit; rrpare, use of parecoxib in postanesthesia care unit; surg, duration of surgery; type of sx, type of operation

Opioid requirement was grouped into no requirement, requirement > 0 to 5 mg MME, and > 5 mg MME. Distribution of ABO blood group and of other potential predictors of opioid requirement were compared across these 3 groups using tabulation and Chi-Square or Fisher’s Exact test as appropriate. Subsequently, multinomial logistic regression was used to evaluate the evidence for any association between the level of opioid requirement and blood group and to identify other variables that showed an association with opioid requirement. All models were controlled for potential confounders as indicated in the Directed Acyclic Graph (DAG) (Fig. 1).

## Results

There were 3316 adult patients enrolled into this study. We divided the recruited population into 3 groups according to the amount of opioid requirement measured as morphine milligram equivalent (MME) in the PACU; group 0 mg when no opioid was required, group > 0–5 mg when the requirement of opioids was more than 0 mg but did not exceed 5 mg and group ≥5 when opioid requirement was at least 5 mg. The demographic data of the 3 groups are presented in Table 1. The majority of the patients (57%) did not receive opioid in PACU. About one-third of the patients received opioid less than 5 mg (MME) while 11% received more than 5 mg. There were more female patients (59%), age less than 65 years old (76%) and ASA classification of II (72%). Blood group O, A, B and AB accounted for 41, 23, 29 and 7%. Sixty-two percent underwent general anesthesia while 20% had spinal anesthesia. Approximately 40% were given at least 5 mg MME intraoperatively. Very few received intraoperative paracetamol (2%) or parecoxib (7%). Almost 3/4 (72.4%) reported VNRS of 0 upon PACU arrival while 11.2, 11.4 and 5% had mild, moderate and severe pain, respectively.

**Table 1:** Demographic data of participants according to their amount of opioid requirement in postanesthesia care unit, based on milligram morphine equivalent

Items	MME 0 mg	MME > 0–5 mg	MME > 5 mg	*P*-value
Total patients (N, %)	1890 (57.0)	1060 (32.0)	366 (11.0)	
*Preoperative Data*				
Sex				< 0.001
Male	816 (60.3)	379 (28.0)	158 (11.7)	
Female	1074 (54.7)	681 (34.7)	208 (10.6)	
Age (years old), median (IQR)	52 (37, 64)	50 (37, 62)	50 (38, 64)	0.20
Age (years old)				0.095
< 65	1426 (56.2)	836 (32.9)	276 (10.9)	
≥ 65	464 (59.6)	224 (28.8)	90 (11.6)	
Religion				0.626
Buddhist	1657 (56.6)	945 (32.3)	328 (11.2)	
Muslim	228 (60.6)	111 (29.5)	37 (9.8)	
Christian	5 (50.0)	4 (40.0)	1 (10.0)	
ASA classification (N, %)				0.068
I	141 (59.2)	75 (31.5)	22 (9.3)	
II	1383 (58.0)	753 (31.6)	250 (10.5)	
III	366 (52.9)	232 (33.5)	94 (13.6)	
BMI (kg/m^2^), median (IQR)	24.4 (21.5, 27.6)	24 (21, 27.7)	24.1 (20.8, 27.1)	0.122
BMI (kg/m^2^), (N, %)				< 0.001
< 25	1042 (55.5)	628 (33.4)	209 (11.1)	
25–34.99	769 (60.9)	362 (28.7)	132 (10.4)	
≥ 35	79 (45.4)	70 (40.2)	25 (14.4)	
Status				0.029
In-patients	1831 (56.6)	1041 (32.2)	361 (11.2)	
Out-patients	59 (71.1)	19 (22.9)	5 (6.0)	
ABO blood group				0.252
O	706 (56.8)	404 (32.5)	133 (10.7)	
A	397 (57.0)	204 (29.3)	96 (13.8)	
B	492 (56.2)	286 (32.6)	98 (11.2)	
AB	119 (54.3)	79 (36.1)	21 (9.6)	
Smoking				0.478
Yes	441 (58.9)	227 (30.3)	81 (10.8)	
No	1449 (56.4)	833 (32.5)	285 (11.1)	
Premedication with benzodiazepine				0.89
Yes	217 (55.9)	128 (33.0)	43 (11.1)	
No	1673 (57.1)	932 (31.8)	323 (11.0)	
Preoperative use of gabapentin				0.001
Yes	53 (43.8)	44 (36.4)	24 (19.8)	
No	1837 (57.5)	1016 (31.8)	342 (10.7)	
Preoperative use of pregabalin				0.227
Yes	5 (45.5)	3 (27.3)	3 (27.3)	
No	1885 (57.0)	1057 (32.0)	363 (11.0)	
Preoperative anxiety score				0.009
0	690 (59.3)	375 (32.2)	99 (8.5)	
1–5	832 (56.4)	467 (31.7)	176 (11.9)	
6–10	368 (54.4)	218 (32.2)	91 (13.4)	
Preoperative pain score				0.033
0	1391 (57.9)	771 (32.1)	240 (10.0)	
1–3	214 (57.8)	112 (30.3)	44 (11.9)	
4–6	197 (52.1)	125 (33.1)	56 (14.8)	
7–1	88 (53.0)	52 (31.3)	26 (15.7)	
*Intraoperative Data*				
Anesthetic techniques				< 0.001
GA	942 (46.1)	824 (40.3)	279 (13.6)	
TCI	100 (84.0)	16 (13.5)	3 (2.5)	
Spinal block	612 (91.5)	48 (7.2)	9 (1.3)	
Nerve block	64 (90.1)	6 (8.5)	1 (1.4)	
Combined	136 (36.8)	161 (43.5)	73 (19.7)	
Others	36 (85.7)	5 (11.9)	1 (2.4)	
Amount of intraoperative opioid use (MME)				< 0.001
0	370 (96.4)	13 (3.4)	1 (0.2)	
> 0–5	965 (58.5)	550 (33.3)	136 (8.2)	
≥ 5	555 (43.3)	497 (38.8)	229 (17.9)	
Intraoperative use of paracetamol				0.002
Yes	26 (37.7)	29 (42.0)	14 (20.3)	
No	1864 (57.4)	1031 (31.8)	352 (10.8)	
Intraoperative use of parecoxib				< 0.001
Yes	94 (40.0)	98 (41.7)	43 (18.3)	
No	1796 (58.3)	962 (31.2)	323 (10.5)	
Intraoperative use of ketamine				0.065
Yes	30 (75.0)	8 (20.0)	2 (5.0)	
No	1860 (56.8)	1052 (32.1)	364 (11.1)	
Intraoperative use of ondansetron				< 0.001
Yes	705 (48.8)	585 (40.5)	154 (10.7)	
No	1185 (63.3)	475 (25.4)	212 (11.3)	
Duration of anesthesia (mins), (median, IQR)	130 (80, 180)	160 (110, 250)	187.5 (135, 278.8)	< 0.001
Duration of surgery (mins), (median, IQR)	75 (40, 125)	117.5 (70, 200)	140 (95, 230)	< 0.001
Type of operation				< 0.001
General surgery	331 (41.2)	360 (44.8)	112 (14.0)	
C-section	127 (91.3)	8 (5.8)	4 (2.9)	
Otolaryngology	263 (62.5)	141 (33.5)	17 (4.0)	
Gynecology	231 (46.3)	199 (39.9)	69 (13.8)	
Neurosurgery	34 (61.8)	19 (34.6)	2 (3.6)	
Obstetrics	59 (68.6)	21 (24.4)	6 (7.0)	
Ophthalmology	100 (74.1)	33 (24.4)	2 (1.5)	
Orthopedics	281 (61.9)	113 (24.9)	60 (13.2)	
Plastic	77 (47.5)	61 (37.7)	24 (14.8)	
Thoracic	6 (17.6)	9 (26.5)	19 (55.9)	
Urological	186 (64.1)	57 (19.7)	47 (16.2)	
Vascular	85 (75.2)	24 (21.2)	4 (3.6)	
Others	110 (88.0)	15 (12.0)	0 (0.0)	
Reoperation				0.123
Yes	44 (46.8)	38 (40.4)	12 (12.8)	
No	1846 (57.3)	1022 (31.7)	354 (11.0)	
Blood loss (ml), (median, IQR)	20 (5150)	50 (10,200)	150 (30,387.5)	< 0.001
*PACU Data*				
Pain score upon PACU arrival				0.033
0	1391 (57.9)	771 (32.1)	240 (10.0)	
1–3	214 (57.8)	112 (30.3)	44 (11.9)	
4–6	197 (52.1)	125 (33.1)	56 (14.8)	
7–10	88 (53.0)	52 (31.3)	26 (15.7)	
Use of paracetamol in PACU				< 0.001
Yes	21 (26.2)	24 (30.0)	35 (43.8)	
No	1869 (57.8)	1036 (32.0)	331 (10.2)	
Use of parecoxib in PACU				< 0.001
Yes	18 (17.6)	37 (36.3)	47 (46.1)	
No	1872 (58.2)	1023 (31.8)	319 (9.9)	
Length of PACU stay (mins) (median, IQR)	45 (30,60)	50 (40,60)	70 (60,98.8)	< 0.001

*Abbreviations*: *MME* Morphine milligram equivalent, *ASA* American Society of Anesthesiologists, *BMI* Body mass index, *GA* General anesthesia, *TCI* Target-controlled infusion, *C-section* Cesarean section, *PACU* Postanesthesia care unit

Fig. 1 demonstrates the Directed Acyclic Graph (DAG) of this study. (DAG can be assumed as a type of flowchart that visualizes a whole causal etiological network, linking causes and effects. Each circle is recognized as a “vertex” and each line is recognized as an “edge.” “Directed” means that each edge has a clear direction, so each edge essentially represents a single directional flow from one vertex to another. “Acyclic” means that there are no loops in the graph. For each vertex, there is no path in the graph that edges get back to that initial vertex.)

Table 2 shows 27 factors included in the DAG analysis. Among these variables, there were 17 statistically significant factors in relation to the amount of opioids required in the PACU. Female required more opioids in the range of > 0–5 mg in comparison to 0 mg (P-value < 0.001). Patients with BMI less than 25 kg/m^2^ or greater than 35 kg/m^2^ required more opioids in the range of > 0–5 mg and > 5 mg compared to 0 mg (*P*-value < 0.001). Outpatients required less opioids in comparison to inpatients (*P* value = 0.02). Those who had been on gabapentin preoperatively demonstrated higher demands for opioids in PACU in comparison to those who had not taken it (*P* value = 0.002). Preoperative anxiety score was significantly related to opioid consumption in immediate postoperative period (*P* value = 0.008). The higher the preoperative anxiety score, the higher the dose of opioids consumed.

**Table 2:** Factors associated with amount of opioid requirement in postanesthesia care unit as indicated by the directed acyclic graph (*N* = 3316)

Factors	Adjustment set	Levels	RRR (95% CI)			*P*-value
			MME > 0–5 mg vs	MME > 5 mg vs	MME > 5 mg vs	
			MME 0 mg	MME 0 mg	MME > 0–5	
Sex	-	Male	1	1	1	< 0.001*
		Female	1.36 (1.17, 1.59)	1.00 (0.80, 1.25)	0.73 (0.58, 0.93)	
Age	-	< 65 years old	1	1	1	0.09
		≥ 65 years old	0.82 (0.69, 0.99)	1.00 (0.77, 1.30)	1.21 (0.92, 1.61)	
Religion						0.62
Buddhist		1	1	1	1	
Muslim		0.85 (0.67, 1.08)	0.82 (0.57,1.18)	0.96 (0.65, 1.42)	0.96 (0.65, 1.42)	
Christian		1.40 (0.38, 5.24)	1.01 (0.12, 8.68)	0.72 (0.08, 6.47)	0.72 (0.08, 6.47)	
ASA	Age, BMI, smoking	ASA I	1	1	1	0.19
		ASA II	1.14 (0.85, 1.54)	1.22 (0.76, 1.97)	1.07 (0.64, 1.77)	
		ASA III	1.27 (0.90, 1.80)	1.66 (0.97, 2.82)	1.30 (0.74, 2.29)	
BMI	-	25 - < 35 kg/m^2^	1	1	1	< 0.001*
		< 25 kg/m^2^	1.28 (1.09, 1.50)	1.17 (0.92, 1.48)	0.91 (0.71, 1.18)	
		≥ 35 kg/m^2^	1.88 (1.33, 2.66)	1.84 (1.13, 3.00)	0.98 (0.60, 1.61)	
Status	-	In-patient	1	1	1	0.02*
		Out-patient	0.57 (0.34, 0.96)	0.43 (0.17, 1.08)	0.76 (0.28, 2.05)	
ABO blood group	-	O	1	1	1	0.26
		A	0.90 (0.73, 1.11)	1.28 (0.96, 1.72)	1.43 (1.05, 1.95)	
		B	1.02 (0.84, 1.23)	1.06 (0.79, 1.41)	1.04 (0.77, 1.41)	
		AB	1.16 (0.85, 1.58)	0.94 (0.57, 1.54)	0.81 (0.48, 1.36)	
Smoking	-	No	1	1	1	0.48
		Yes	0.90 (0.75, 1.07)	0.93 (0.71, 1.22)	1.04 (0.78, 1.39)	
Premedication with benzodiazepine	-	No	1	1	1	0.89
		Yes	1.06 (0.84, 1.34)	1.03 (0.72, 1.45)	1.03 (0.71, 1.49)	
Preoperative use of gabapentin	-	No	1	1	1	0.002*
		Yes	1.50 (1.00, 2.26)	2.43 (1.48, 4.00)	1.62 (0.97, 2.70)	
Preoperative use of pregabalin	-	No	1	1	1	0.33
		Yes	1.07 (0.26, 4.49)	3.12 (0.74, 13.09)	2.91 (0.58, 14.49)	
Preoperative anxiety score	-	0	1	1	1	0.008*
		1–5	0.99 (0.83, 1.18)	1.47 (1.12, 1.92)	1.48 (1.11, 1.96)	
		6–10	1.02 (0.82, 1.26)	1.74 (1.26, 2.39)	1.69 (1.21, 2.37)	
Preoperative pain score	Sex, age, ABO blood group, benzodiazepine, gabapentin, pregabalin	0	1	1	1	0.24
		1–3	0.94 (0.74, 1.21)	1.13 (0.79, 1.62)	1.20 (0.82, 1.76)	
		4–6	1.12 (0.87, 1.43)	1.51 (1.08, 2.10)	1.35 (0.94, 1.92)	
		7–10	1.03 (0.72, 1.48)	1.51 (0.94, 2.41)	1.46 (0.88, 2.42)	
Anesthetic technique	-	GA	1	1	1	< 0.001*
		TCI	0.18 (0.11, 0.31)	0.10 (0.03, 0.32)	0.55 (0.16, 1.91)	
		Spinal block	0.09 (0.06, 0.12)	0.05 (0.02, 0.10)	0.55 (0.27, 1.14)	
		Nerve block	0.11 (0.05, 0.25)	0.05 (0.01, 0.38)	0.49 (0.06, 4.11)	
		Combined	1.35 (1.06, 1.73)	1.81 (1.32, 2.48)	1.34 (0.98, 1.82)	
		Others	0.16 (0.06, 0.41)	0.09 (0.01, 0.69)	0.59 (0.07, 5.08)	
Amount of intraoperative opioid use (MME)	Age, ASA, BMI, anesthetic technique, duration of anesthesia, duration of surgery, type of operation, reoperation, blood loss	-	1.01 (1.00, 1.02)	1.01 (1.00, 1.03)	1.00 (1.00, 1.01)	0.02*
Intraoperative use of paracetamol	-	No	1	1	1	0.003*
		Yes	2.02 (1.18, 3.44)	2.85 (1.47, 5.51)	1.41 (0.74, 2.71)	
Intraoperative use of parecoxib	-	No	1	1	1	< 0.001*
		Yes	1.95 (1.45, 2.61)	2.54 (1.74, 3.72)	1.31 (0.89, 1.91)	
Intraoperative use of ketamine	-	No	1	1	1	0.053
		Yes	0.47 (0.22, 1.03)	0.34 (0.08, 1.43)	0.72 (0.15, 3.42)	
Intraoperative use of ondansetron	-	No	1	1	1	< 0.001*
		Yes	2.07 (1.78, 2.41)	1.22 (0.97, 1.53)	0.59 (0.46, 0.75)	
Duration of anesthesia	Duration of surgery	-	1.00 (1.00, 1.00)	1.00 (0.99, 1.00)	1.00 (0.99, 1.00)	0.02*
Duration of surgery	-	-	1.00 (1.00, 1.01)	1.01 (1.00, 1.01)	1.00 (1.00, 1.00)	< 0.001*
Type of operation	-	General surgery	1	1	1	< 0.001*
		C-section	0.06 (0.03, 0.12)	0.09 (0.03, 0.26)	1.61 (0.48, 5.44)	
		Otolaryngology	0.49 (0.38, 0.63)	0.19 (0.11, 0.33)	0.39 (0.22, 0.67)	
		Gynecology	0.79 (0.62, 1.01)	0.88 (0.62, 1.24)	1.11 (0.79, 1.58)	
		Neurosurgery	0.51 (0.29, 0.92)	0.17 (0.04, 0.74)	0.34 (0.08, 1.48)	
		Obstetrics	0.33 (0.19, 0.55)	0.30 (0.13, 0.72)	0.92 (0.36, 2.33)	
		Ophthalmology	0.30 (0.20, 0.46)	0.06 (0.01, 0.24)	0.19 (0.05, 0.82)	
		Orthopedics	0.37 (0.28, 0.48)	0.63 (0.44, 0.90)	1.71 (1.17, 2.49)	
		Plastic	0.73 (0.50, 1.05)	0.92 (0.56, 1.53)	1.26 (0.75, 2.12)	
		Thoracic	1.38 (0.48, 3.92)	9.36 (3.65, 24.02)	6.78 (2.98, 15.42)	
		Urological	0.28 (0.20, 0.39)	0.75 (0.51, 1.10)	2.65 (1.70, 4.12)	
		Vascular	0.26 (0.16, 0.42)	0.14 (0.05, 0.39)	0.54 (0.18, 1.58)	
Reoperation	-	No	1	1	1	0.13
		Yes	1.56 (1.00, 2.42)	1.42 (0.74, 2.72)	0.91 (0.47, 1.76)	
Blood loss	Type of operation	Based on 1 ml	1.00 (1.00, 1.00)	1.00 (1.00, 1.00)	1.00 (1.00, 1.00)	< 0.001*
		Based on 10 ml	1.09 (1.06, 1.12)	1.15 (1.11, 1.18)	1.05 (1.02, 1.08)	< 0.001*
Pain score upon PACU arrival (model 1)	ABO, age, anesthetic technique, benzodiazepine, blood loss, BMI, duration of anesthesia, intraoperative use of parecoxib, gabapentin, amount of intraoperative opioid use (MME), intraoperative use of ketamine, intraoperative use of paracetamol, preoperative anxiety, pregabalin, preoperative pain score, status, religious, reoperation, sex, smoking, duration of surgery, type of operation	-	2.09 (1.98, 2.21)	3.03 (2.79, 3.30)	1.45 (1.36, 1.55)	< 0.001*
Pain score upon PACU arrival (model 2)	Age, anesthetic technique, ASA, benzodiazepine, BMI, intraoperative use of parecoxib, gabapentin, amount of intraoperative opioid use (MME), intraoperative use of ketamine, intraoperative use of paracetamol, preoperative anxiety, pregabalin, preoperative pain score, religious, reoperation, sex, smoking, duration of surgery, type of operation	-	2.09 (1.98, 2.21)	3.04 (2.79, 3.30)	1.45 (1.36, 1.55)	< 0.001*
Use of paracetamol in PACU	-	No	1	1	1	< 0.001*
		Yes	2.06 (1.14, 3.72)	9.41 (5.41, 16.37)	4.56 (2.68, 7.78)	
Use of parecoxib in PACU	-	No	1	1	1	< 0.001*
		Yes	3.76 (2.13, 6.64)	15.32 (8.79, 26.72)	4.07 (2.60, 6.38)	

*Abbreviations*: *RRR* Relative risk ratio, *CI* Confident interval, *MME* Morphine milligram equivalent, *ASA* American Society of Anesthesiologists, *BMI* Body mass index, *GA* General anesthesia, *TCI* Target-controlled infusion, *C-section* Cesarean section, *PACU* Postanesthesia care unit

Regarding anesthetic techniques, in comparison to general anesthesia - all techniques (TCI, spinal block, nerve block and others) were related with less opioid consumption in PACU except combined general and regional anesthesia technique (*P*-value < 0.001). Patients receiving higher intraoperative opioids required higher dose of opioids postoperatively (*P* value = 0.02). Those who were given intraoperative paracetamol, parecoxib and ondansetron demonstrated higher opioid consumption in PACU compared to those who did not receive these drugs (*P* values = 0.003, < 0.001 and <  0.001, respectively). Patients receiving intraoperative ketamine had a non-statistically significant trend for less opioid consumption in comparison to those not receiving ketamine intraoperatively (*P* value = 0.053). Longer duration of anesthesia and surgery were related with higher amount of opioids required in PACU (*P* values = 0.02 and <  0.001, respectively).

Regarding type of operation, in comparison to general surgery - other types of surgical procedures showed less opioid consumption in PACU except thoracic surgery which showed higher opioid requirement in all ranges while C-section, gynecological, orthopedic, urological and plastic surgeries demonstrated higher opioid consumption in the range of > 5 mg in comparison to > 0–5 mg (*P*-value < 0.001). Amount of blood loss was directly correlated with opioid requirement in PACU (*P*-value < 0.001). Pain score upon PACU arrival with either model 1 or 2 was directly correlated with immediate postoperative opioid consumption (*P*-value < 0.001). Patients receiving either paracetamol or parecoxib in PACU demonstrated higher opioid consumption (*P*-value < 0.001). ABO blood group was not related to the amount of opioids required in PACU.

## Discussion

We have found many factors related to the amount of opioids required (as MME) in PACU. However, in our study, ABO blood group was not found to influence the amount of opioid consumption in PACU. The ABO blood system is inherited by genes on chromosome 9. There are 4 major types of blood: O, A, B, and AB. Specific sugars on surface of erythrocytes determine blood grouping, N-acetylgalactosamine for A antigen and D-galactose for B antigen. These 2 sugars are built upon H antigen. In people with unmodified H antigen, their blood group is O because both A and B antigens can’t adhere to erythrocytes [14].

Genetic factors such as gender and hair color have been demonstrated to have an influence on pain sensitivity and modulation [15, 16]. It has been reviewed that genetic variants in mu opioid receptor, voltage-gated channel alpha subunit 11 (SCN11A), brain-derived neurotrophic factor (BDNF) and catechol-O-methyltransferase (COMT) genes are related to inter-individual variability in postoperative pain severity and analgesic response. However, to accurately identify patients with predisposed genetic for severe postoperative pain or development of chronic postoperative pain, is still impossible [17].

In this study, we focused on the association between ABO blood group and early postoperative opioid consumption in PACU. Jasim et al. reported that blood group O was one of the factors affecting pain following cesarean section. However, this study did not include data on an amount of opioid requirement [11]. Nimmaanrat et al., did a retrospective study in 1530 patients undergoing cesarean delivery and found that ABO blood group was not a predictive factor for the quantity of opioid consumption within the first 24 h after surgery [13]. Similarly, ABO blood group did not significantly affect consumption of postoperative analgesics in patients undergoing anterior cruciate ligament reconstruction [12]. Pain is defined as an unpleasant sensory and emotional experience associated with, or resembling that associated with, actual or potential tissue damage. Multiple factors including biological, psychological and social aspects affect each individual’s experience of pain [18]. As pain is multifactorial, single entity such as ABO blood group may not have a significant effect on pain severity and analgesic demand, especially in clinical setting rather than experimental setting. Further studies are warranted to identify the correlation between ABO blood group and postoperative pain, quantity of analgesic use, analgesic response and risk of developing chronic postsurgical pain.

Female sex, BMI < 25 and > 35 kg/m^2^, preoperative use of gabapentin, preoperative anxiety score, amount of intraoperative opioids used, intraoperative use of paracetamol, parecoxib and ondansetron, duration of anesthesia and surgery, amount of blood loss, pain score upon PACU arrival, and use of paracetamol and parecoxib in immediately postoperative period were positively linearly associated with the amount of opioid requirement in PACU. On the other hand, outpatient was negatively linearly related with the amount of PACU opioid consumption. Patients undergoing thoracic surgery or those having combined general and regional anesthesia demonstrated higher demand for postoperative opioids, while patients receiving TCI, spinal or nerve block as the primary anesthetic technique had less opiate consumption.

Our finding showed that female patients required higher opioids in PACU. Female sex was previously found as a risk factor for increased postoperative pain on PACU admittance [19] requiring a higher fentanyl dose [20]. Sex hormones and receptors play a role on complex pain sensitivity for both pro-nociceptive and anti-nociceptive effects [21]. We found that higher BMI is associated with higher opioid requirement in PACU. Increased BMI was previously demonstrated as an independent risk factor for moderate to severe pain within the first 24 h after surgery [22]. However, it was also reported that BMI of patients undergoing ankle fracture surgery, was not associated with opioid requirement in PACU [23]. In this study, we found that outpatient required less opioids in PACU. This is straightforward as procedures performed in this group of patients are less invasive than the majority of procedures done in inpatients.

Interestingly, our study found that patients who had been on gabapentin preoperatively required more opioids in PACU. Gabapentin is an anticonvulsant with anti-neuropathic pain property [24]. Patients with gabapentin prior to surgery were those with neuropathic pain. This kind of patients may have higher pain sensitivity due to pre-existing central and peripheral sensitization [25]. We found that the higher the preoperative anxiety score, the higher the dose of consumed opioid in PACU. High state anxiety score was previously demonstrated as independently associated with more pethidine requirement following C-section [26]. On the other hand, another study revealed that preoperative anxiety did not correlate with augmented postoperative opioid use [27].

We have found that combined anesthetic technique was correlated with higher opioid consumption in PACU. In general, we do combined technique in major surgical cases with extensive tissue injury as this may partially explain why these patients needed more opioids in the early postoperative period. We found that thoracic surgery was a procedure with the highest opioid consumption in PACU. Pain following thoracic surgery is severe and represents one of the most severe of all surgeries. Surgical incision, muscle and ligament manipulation, compressive retraction of ribs, possible rib fractures, irritation of pleura and chest tubes lead to significant postoperative pain [28]. We revealed that the amount of intraoperative opioid administration was correlated with the amount of opioid requirement in PACU. On the other hand, Dahmani et al. found that amount of intraoperative opioid was not an independent predictive factor of early morphine requirement in PACU [5].

It is interesting that in this study, patients receiving either intraoperative paracetamol nor parecoxib showed higher opioid consumption in PACU. A Cochrane review revealed that a single dose of intravenous paracetamol provides about 4 h of effective analgesia in approximately 36% of acute postoperative pain patients [29]. In regard to parecoxib, a Cochrane review demonstrated that a single dose of parecoxib provides effective analgesia for 50–60% of those treated, in comparison to around 15% with placebo [30]. According to financial restriction, we did not use multimodal analgesia in all patients. We utilized parenteral paracetamol and parecoxib in only cases with massive tissue injury so they tended to have higher pain and demand for opioids in PACU.

We straightforwardly found that duration of anesthesia and surgery as well as the amount of blood loss were related to the amount of opioids required in PACU. Longer duration of surgery than 2 h was previously reported as a factor affecting pain in PACU [31]. Our finding was also straightforward that severity of pain upon PACU arrival indicated the amount of opioids consumed in PACU. In fact, administration of paracetamol and parecoxib in PACU should reduce the amount of opioids used. But in our practice, we usually gave opioids first before considering giving paracetamol and parecoxib as per financial restriction.

The strengths of this study are its prospective design with a large number of patients. However, it included all types of anesthetic techniques and operations so its main weakness is heterogeneity of studied population. Including various kinds of surgical patients undergoing various types of anesthetic procedures into our study should be considered while interpreting the results as it may pose different influence on opioid consumption in PACU. Although our study reflects norms of ordinary practice with a large variety of patients, anesthetic techniques and surgical procedures, applying its findings needs careful consideration and interpretation. We did not collect data on preoperative long-term use of opioids and benzodiazepines which might affect the amount of opioid requirement in the early postoperative period.

## Conclusion

This prospective cohort study has demonstrated various significant factors to be associated with the amount of opioid consumption in PACU. Some factors can be modified to make patients more comfortable in the initial postoperative period. However, ABO blood group is not identified as a factor affecting early opioid requirement in PACU.

## Data Availability

The datasets used and/or analyzed during the current study are available from the corresponding author on reasonable request.
